# Nanoparticle architecture preserves magnetic properties during coating to enable robust multi-modal functionality

**DOI:** 10.1038/s41598-018-29711-0

**Published:** 2018-08-23

**Authors:** Lauren E. Woodard, Cindi L. Dennis, Julie A. Borchers, Anilchandra Attaluri, Esteban Velarde, Charlene Dawidczyk, Peter C. Searson, Martin G. Pomper, Robert Ivkov

**Affiliations:** 1 Institute for NanoBioTechnology, Johns Hopkins University, Baltimore, MD 21218 USA; 2 Material Measurement Laboratory, NIST, Gaithersburg, MD 20899-8550 USA; 3 NIST Center for Neutron Research, NIST, Gaithersburg, MD 20899-6102 USA; 4 Department of Radiation Oncology and Molecular Radiation Sciences, Johns Hopkins University School of Medicine, Baltimore, MD 21231 USA; 5 Department of Oncology, Johns Hopkins University School of Medicine, Baltimore, MD 21231 USA; 6 Department of Materials Science and Engineering, Johns Hopkins University, Baltimore, MD 21218 USA; 7 Division of Cancer Imaging Research, Russell H. Morgan Department of Radiology and Radiological Sciences, Johns Hopkins University School of Medicine, Baltimore, MD 21231 USA; 8 Department of Mechanical Engineering, Johns Hopkins University, Baltimore, MD 21218 USA; 9 Present Address: Department of Mechanical Engineering, School of Science, Engineering, and Technology, Pennsylvania State University, Harrisburg,Middletown, PA 17057 USA

## Abstract

Magnetic iron oxide nanoparticles (MIONs) have established a niche as a nanomedicine platform for diagnosis and therapy, but they present a challenging surface for ligand functionalization which limits their applications. On the other hand, coating MIONs with another material such as gold to enhance these attachments introduces other complications. Incomplete coating may expose portions of the iron oxide core, or the coating process may alter their magnetic properties. We describe synthesis and characterization of iron oxide/silica/gold core-shell nanoparticles to elucidate the effects of a silica-gold coating process and its impact on the resulting performance. In particular, small angle neutron scattering reveals silica intercalates between iron oxide crystallites that form the dense core, likely preserving the magnetic properties while enabling formation of a continuous gold shell. The synthesized silica-gold-coated MIONs demonstrate magnetic heating properties consistent with the original iron oxide core, with added x-ray contrast for imaging and laser heating.

## Introduction

The increasing availability of nanostructured materials having controllable magnetic properties has created widespread interest in developing nanometer-size particles for medical diagnosis and therapy. Few of these proposed constructions, however, have established a role in medicine through human clinical trials and regulatory approval^1,2^. A noteworthy exception is magnetic iron oxide nanoparticle (MION) formulations that have enjoyed *in vivo* human use in clinical settings for several decades. MIONs have demonstrated effectiveness and safety^3^ for a variety of medical applications that include enhanced contrast for magnetic resonance imaging (MRI)^2,4,5^, intravenous anemia therapy^6^, and hyperthermia therapy for glioblastoma^1,7^. They are thus the subject of considerable research effort to develop multifunctional capabilities.

MIONs can generate heat via hysteresis losses when they are exposed to an alternating magnetic field (AMF)^8–14^. Coupled with their inherent MRI contrast, they provide a natural ‘theranostic’ platform for disease treatment and diagnosis. MIONs and the heat they can generate were incorporated in the developing clinical scenario for imaging-guided palliative treatments of late-stage liver cancer^15–20^. Image-guided interventional procedures for drug delivery form the front-line of therapy for some cancers^21,22^; but, while initially promising, imaging guidance to ‘target’ MION deposition and subsequent heat therapy fell short^8,20^. Though arguably one of the first nanoparticle demonstrations of theranostics, the MION formulations then available produced low hysteresis loss power heating, necessitating high tissue concentrations which proved unachievable and produced significant MRI (magnetic susceptibility) artifacts. X-ray computed tomography (CT) imaging was also pursued to provide the imaging guidance, but the available biocompatible aqueous suspensions of MIONs lacked sufficient x-ray opacity to provide meaningful contrast^20^. It has thus been acknowledged for some time that a nanoparticle construct providing MRI contrast (at low tissue concentrations) and x-ray CT contrast (at high concentrations) with therapy (heating) would enhance and simplify targeted delivery of agents for diagnosis, imaging-guided treatment, and post-treatment follow-up^23^. Such a construction could also potentially provide molecular targeting if it possesses a non-toxic, chemically inert surface that enables facile chemistry for biofunctionalization^5,23–28^.

The motivation to pursue development of multi-functional capabilities using MIONs has been clear for decades, yet materials chemistry challenges have impeded meaningful progress. MIONs typically comprise a combination of magnetite (Fe_3_O_4_) or maghemite (γ-Fe_2_O_3_), which are hydrophobic making them unstable in biological media. Other iron oxides also form during synthesis, from many possible side reactions, to contaminate the particle surface^8,28–31^. The complex surface chemistry of MIONs also limits reliable functionalization with organic and biological molecules. It is possible, however, to exploit facile covalent biofunctionalization using thiol chemistry which is made possible by addition of a gold coating layer to the MIONs^23,28,30^. An added benefit is the potential of gold-coated core-shell nanoparticles to be high contrast agents for x-ray CT imaging^23,24^. A complete gold coating can also impart optical contrast to MION-Au nanocomposite structures via plasmon resonance^23,24,28,30,32–36^. While prior efforts to produce gold-coated iron oxide nanoparticles have yielded constructs that combine optical or x-ray contrast with MRI capability^23,28,34,35,37^, the ultimate goal is to develop a theranostic (dual MRI/CT imaging capability, hyperthermia, and biofunctionalization) iron oxide-gold core-shell nanoparticles^23,24,38–41^. No formulations developed to date have demonstrated combined x-ray and magnetic imaging properties within a single nanoparticle construction that also provides heating with magnetic fields and light.

Gold-coated MIONs that provide MRI and CT contrast while preserving magnetic and optical heating are difficult to construct because the incompatible surface energies of these materials inhibits complete coating of magnetite by gold^42^. An incomplete coating leaves portions of the iron oxide core exposed to disrupt biofunctionalization. It can also scatter light or disrupt surface plasmon resonance degrading optical applications^28,42^. The coating process can also alter the surface structure to change the composition and magnetic properties, potentially reducing magnetic heating and imaging performance^8,43^. Sood, *et al*. recently noted that magnetization saturation (*M*_*s*_) of iron oxide nanoparticles decreases from 42.81 to 3.54 A-m^2^/kg (emu/g) when coated with gold^44^. One possible solution to achieve coating is to incorporate an intermediate layer^28,42^. Among the intermediate layers that have been explored for gold and iron oxide with varying degrees of success are silica, ionic surfactants, biological or synthetic organic polymers, or combinations of various organic and inorganic agents that served to stabilize the iron oxide in the suspending medium and to enhance gold adhesion^25,30,44–47^. The functionalities of these heterogeneous nanoparticle composites vary greatly due to significant differences in the characteristics of the interfacial structure on the nanoscale. Therefore, it is critical to understand, accurately and completely, what structural and magnetic changes result from coating the MIONs, to aid optimization of synthesis methods and to validate the resulting product for its intended end-use.

Here, we report results obtained from a multi-step synthesis procedure to coat MIONs with a silica layer before addition of the gold layer. The primary objective was to obtain a gold-coated iron oxide formulation that provided x-ray contrast while retaining magnetic hysteresis heating, and to characterize this construct with small angle neutron scattering (SANS). The MIONs comprised a dense polycrystalline core and possessed a magnetic core-shell structure leading to hysteresis heating properties useful for magnetic hyperthermia^29,48,49^. Detailed magnetic and structural analysis of the silica- and gold-silica-coated MIONs revealed the MION cores were coated by the silica layer in a manner contrary to current expectations for a dense core. Specifically, silica intercalated between the individual iron oxide crystallites within the dense solid core instead of encapsulating the entire iron oxide polycrystalline core as a single entity. The silica surface of the elliptical composite facilitated formation of a continuous gold shell. Magnetic characterization and heating with alternating magnetic fields confirmed that the original magnetic properties of the MIONs were only modestly altered, presumably because the silica effectively passivated the MION crystallite surfaces limiting further change in subsequent gold precipitation and reduction reactions. MRI and x-ray CT contrast were characterized for the gold-silica-MIONs and were compared with the precursor constructs, confirming the dual-modality imaging capabilities and extending the range of concentration for MION detection. Heating performance with both magnetic fields and laser was characterized, and proof-of-concept *in vivo* imaging and heating of a mouse subcutaneous xenograft model of human prostate cancer were demonstrated.

## Results and Discussion

**Figure 1:**
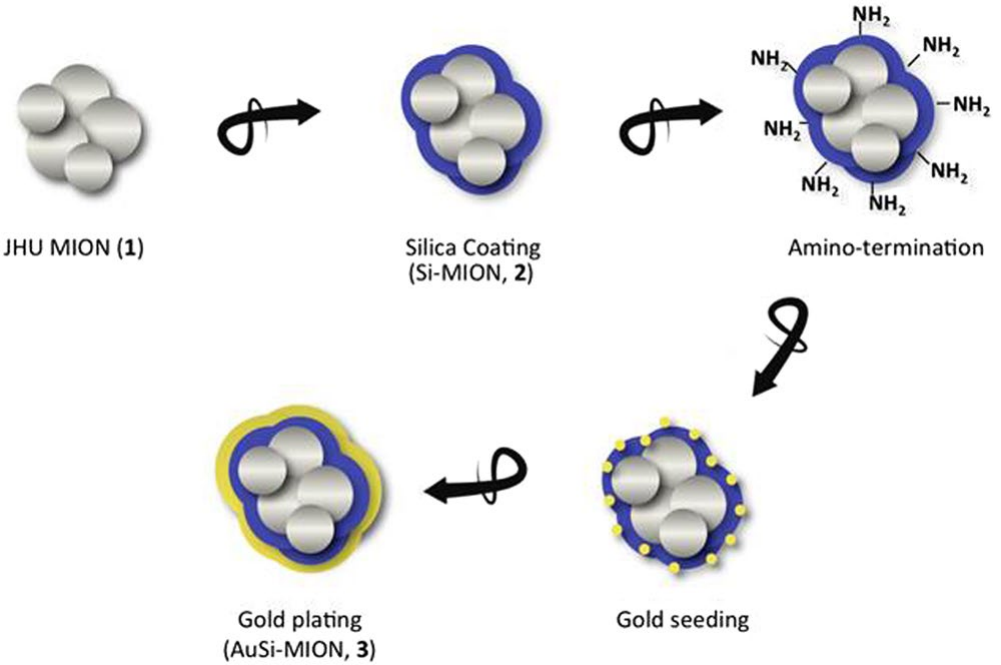
Synthesis schematic of gold-silica-coated MIONs. Iron oxide cores (JHU MIONs, **1**) were coated with silica using tetraethylorthosilicate to form Si-MIONs (**2**). The Si-MIONs were amine-terminated using 3-aminopropyltrimethoxysilane and seeded by a colloidal gold solution containing 1–2 nm gold seeds. Finally, a gold shell was grown on the surface by the reduction of chloroauric acid to form AuSi-MIONs (**3**).

A schematic of the chemistry and coated particle structure, and summary of samples prepared and measurements conducted are provided in Fig. 1, (AuSi-MION, **3**) and in Table 1, respectively.

**Table 1:** Summary of nanoparticle samples used for measurements.

	Concentration (mg Fe/ml solution)	Avg Diameter by DLS (PDI)**	TEM	MRI phantom studies	CT phantom studies	SLP	SAR	*In vivo* CT	*In vivo* hyperthermia	Histology	SQUID	SANS
1) JHU MION	20	55 nm (0.17)	X	X	X	X	X	X	X	X	X	X
2) Si-MION	20	81 nm*** (0.19)	X	X	X	X		X*	X*	X*		
3) AuSi-MION	14	145 nm*** (0.15)	X	X	X	X	X	X	X	X		
4) Si-MION (scale-up)	20	98 nm (0.23)							X	X		
5) AuSi-MION (scale-up)	14	155 nm (0.22)							X	X		

*Data not shown.

**Polydispersity index (PDI) values are shown in parentheses.

***Nanoparticles used for *in vivo* studies were synthesized to maintain a minimum diameter.

**Figure 2:**
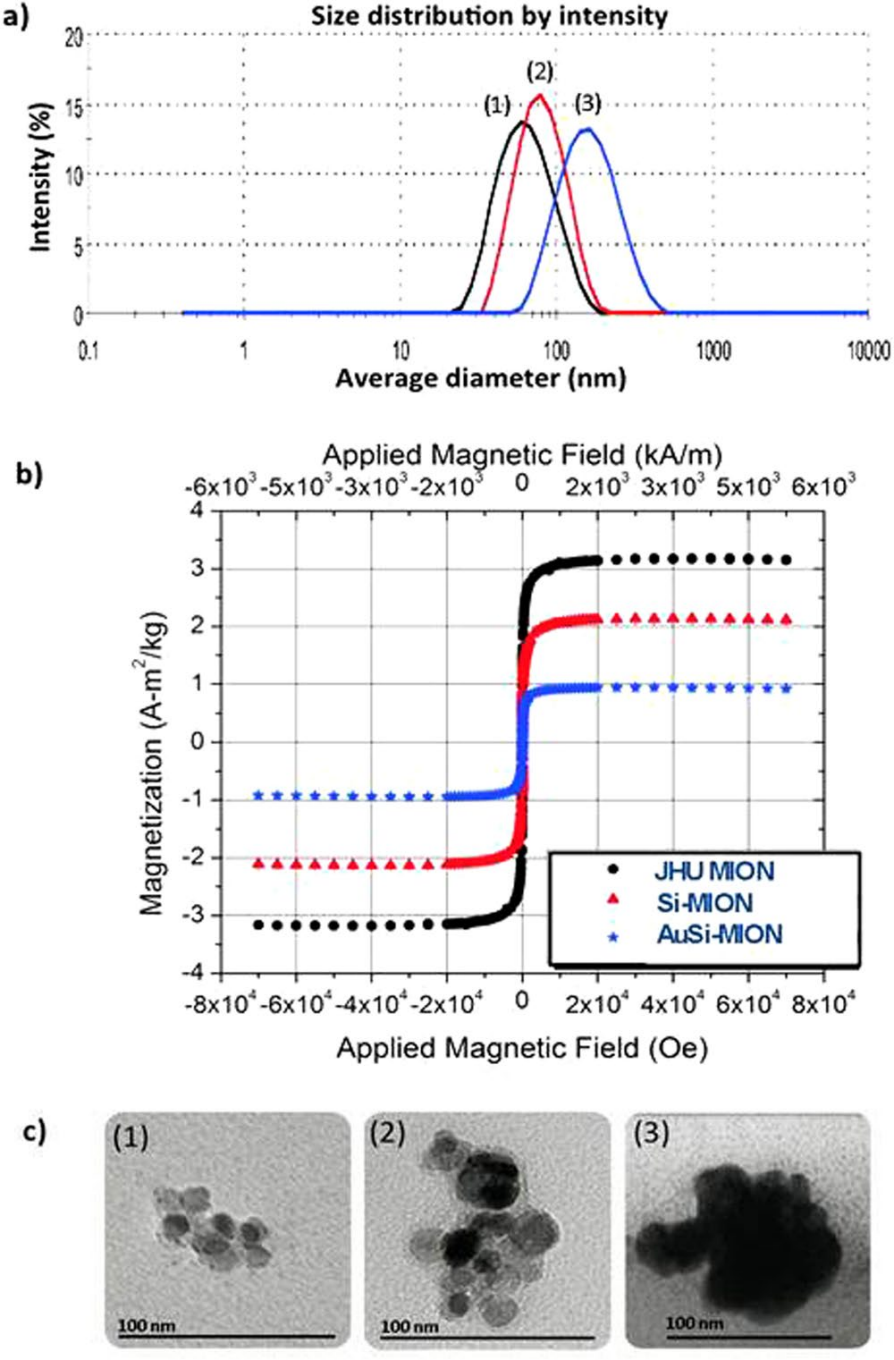
Physical characterization of MIONs. (**a**) Dynamic light scattering (DLS) of (**1**) JHU MIONs – 55 nm, (**2**) Si-MIONs – 81 nm and (**3**) AuSi-MIONs – 145 nm. (**b**) SQUID magnetometry measurements of magnetization of MIONs as a function of external field strength. Data are normalized by total solid content, without removal of the silica and gold contributions. (**c**) Transmission electron microscopy (TEM) of (**1**) JHU MION cores, (**2**) silica-coated MIONs and (**3**) gold and silica-coated MIONs.

**Figure 3:**
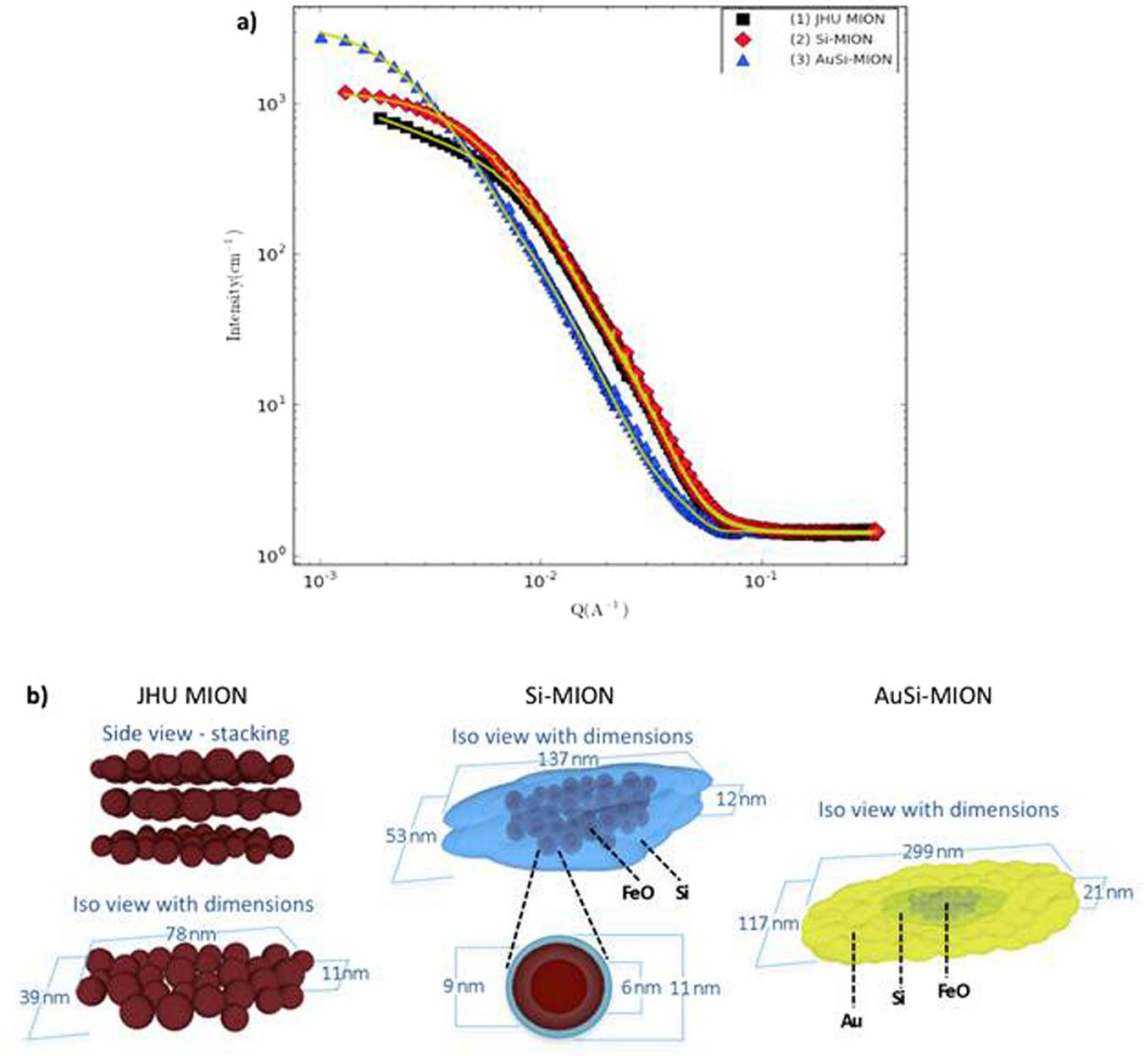
Small angle neutron scattering (SANS) data and analysis of MION size and shape. (**a**) SANS scattering data (points) with correlated model fits (solid lines) obtained using dimensions and 3D geometrical models for JHU MIONs (black squares), Si-MION (red diamonds), and AuSi-MION (blue triangles) as shown in (**b**), respectively and based on SANS analysis. Graphics of nanoparticle constructs are used with permission from A.K. Woodard.

The nanoparticles chosen for this study were citrate-stabilized dense polycrystalline core of magnetic iron oxide, previously described as JHU MIONs (1, Fig. 1)^29,48,49^. JHU MIONs were prepared by high-gravity controlled precipitation^50^ with thermal aging, and stabilized with citric acid^48^. Physical structures of the samples used in the current studies were measured by dynamic light scattering (DLS), transmission electron microscopy (TEM), and small angle neutron scattering (SANS)^51^. The MIONs comprise a stable aqueous suspension of ~55 nm (polydispersity index (PDI) of ~0.17) nanoparticles containing agglomerated iron oxide crystallites (6–11 nm diameter) to form a dense polycrystalline core (Figs 2a,c and 3). As previously reported the dense polycrystalline core of JHU MIONs exhibits an inverse spinel-type structure and comprises a mixture of magnetite (Fe_3_O_4_) and maghemite (ɣ-Fe_2_O_3_), as determined by Mössbauer spectroscopy^29^.

For silica coating, a modified Stöber process was developed to control the thickness of the silica layer by stoichiometric addition of tetraethylorthosilicate (TEOS), and formation of the silica coating was confirmed by TEM (Figs 2a,c and 3)^52^. 3-Aminopropyltrimethoxy-silane (APTMS) was added to the Si-MION (**2**) suspension to form amino-terminated Si-MIONs, which were then seeded with a colloidal suspension of 1–2 nm gold nanoparticles. Chloroauric acid was added to the suspension and reduced with hydroxylamine, initiating gold precipitation onto the seeded surface of the Si-MIONs to form gold shells (AuSi-MIONs **3**, 145 nm, PDI of ~0.15), see Figs 2a,c and 3.

The most interesting observation of MION physical transformation with coating occurs following the initial addition of silica. Small angle neutron scattering (SANS) has proven to be a powerful probe of the internal structural (1 nm to 200 nm) and magnetic properties of nanostructured ensembles that are not readily apparent from bulk analysis^29^. Careful choice of the experimental conditions, i.e. sample (iron oxide nanoparticles) and solvent (H_2_O or D_2_O), enables scattering contrast variations to highlight specific features such as the magnetic scattering, or elemental compositions of individual layers^29^. Here, SANS revealed the coating evolution from a comparison among correlated fitted results for all three nanoparticle constructs (Fig. 3a). Robust solutions to model fitting of SANS data (Fig. 3a,b) were only possible when data from multiple complementary techniques, such as TEM (Fig. 2c) and DLS (Fig. 2a), were combined to identify the combination of SANS geometric models appropriate for each particle construct (See Supporting information for details)^53^. Note that SANS, DLS, and TEM provided no evidence consistent with the formation of pure silica or pure gold nanoparticles.

Fits to SANS data obtained from the uncoated JHU MIONs (Fig. 3a) proved sensitive to the dimensions of a flat ellipsoid model (Fig. 3b), which contains clusters of small iron oxide crystallites (Fig. 2c **(1)**). The individual crystallites within each core were indistinguishable because their scattering length densities (SLDs) are comparable, yielding negligible contrast. TEM and SANS data measured from the Si-MION sample clearly demonstrated formation of a robust silica layer encasing the iron oxide cores (Fig. 2c **(2)** and Fig. 3a), as expected. However, analysis of the SANS data demonstrated that the silica also diffused into the dense polycrystalline core to surround individual iron oxide crystallites (Fig. 2c, part (**2**)). Surprisingly, a geometrical construct consistent with the SANS data (Fig. 3b) comprised clusters of iron oxide crystals that were coated by a layer of mixed iron oxide/silica and then by a thin (~2 nm) layer of silica only. These layers are distinguishable with SANS because silica and iron oxide possess very different SLDs, thus increasing SANS sensitivity to the individual iron oxide crystallites that form the core. The AuSi-MION TEM (Fig. 2c **(3)**) showed a continuous, electron-dense coating covering the entire surface of each particle consistent with a distinct gold shell. Again, SANS models were consistent with elliptical geometries larger than either the precursor intermediates (Fig. 3b), indicating addition of a coating layer, though the local encasement of the iron oxide crystallites with silica was preserved. Use of the shape parameters obtained from SANS fitting enabled reconciliation of differences observed between the DLS and SANS data interpretation. When ellipsoidal objects were considered with interpreting the DLS model, namely that DLS presumes a spherical particle and is most sensitive to the median dimension of an ellipsoid, agreement between DLS and SANS ellipsoid models resulted (see Supplemental information).

Field-dependent magnetization measurements of the JHU MION constructs demonstrated that magnetization saturation (*M*_*s*_) of AuSi-MIONs was reduced to about 30% of the uncoated JHU MIONs *M*_*s*_, when normalized to total solid content (Fig. 2b). This decrease is expected, as the gold and silica provide only a diamagnetic contribution which was not subtracted and the additional mass of the gold and silica are expected to reduce *M*_*s*_ accordingly. An additional contribution to the decreased *M*_*s*_ (see Supplemental Materials) originates from background contributions that cannot be properly accounted because of silica intercalation. Thus, precise comparisons of magnetization among the samples is precluded, however it is possible to extract general features from a comparison. When examining the coercivity at 5 K (see Supplemental Material), there is an initial increase from 24 kA/m for the JHU-MIONs to 32 kA/m for the Si-MIONs, which can be most readily attributed to the rigid encapsulation of the MIONs in silica. After coating with Au, however the coercivity returns to its previous value of 24 kA/m. These results suggest that the Au coating has a modest effect on the magnetic properties of the MIONs.

**Figure 4:**
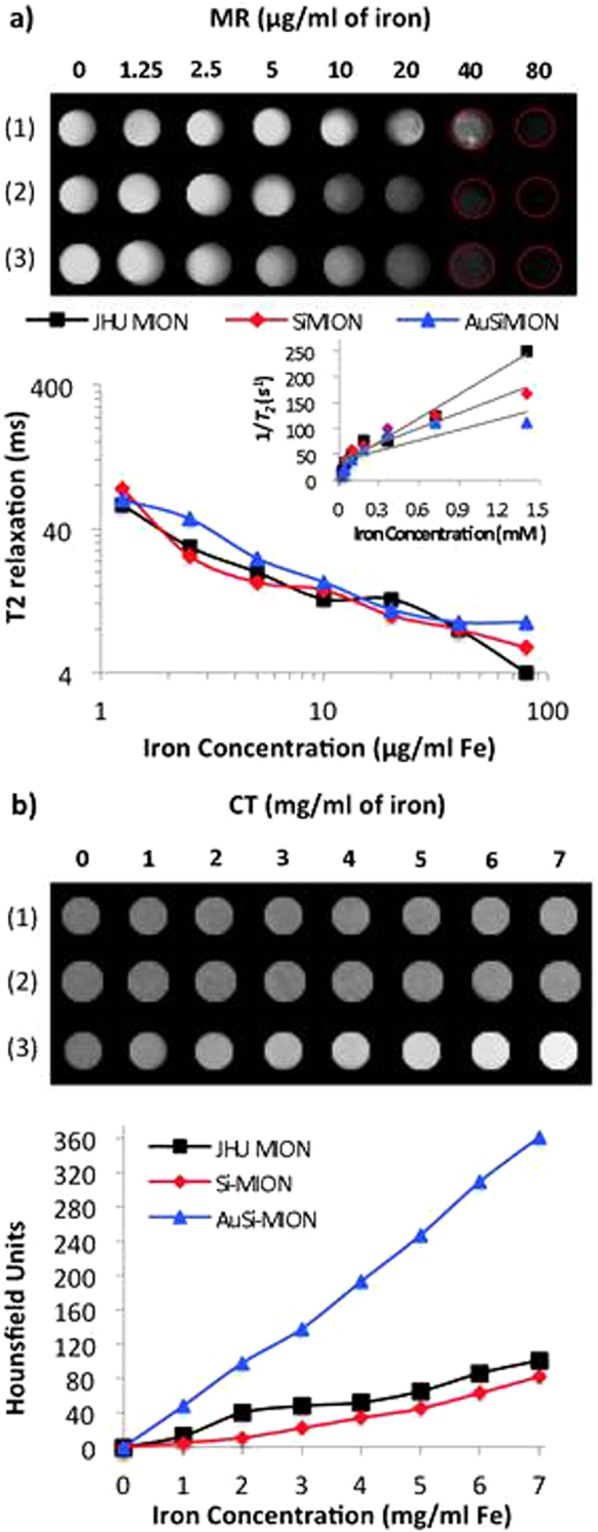
Imaging MRI and CT. (**a**) MR imaging contrast of JHU MIONs (1), Si-MIONs (2) and AuSi-MIONs (3). Imaging of gel phantoms over a range of 0–80 μg/ml (0–1.4 mM) based on iron content, showing *T*_2_ effect as iron concentration increases (top). *T*_2_ relaxation (ms) calculated from spin-echo MR imaging of phantoms (bottom). Inset shows concentration (mM) versus 1/*T*_2_, the slope of which gives transverse relaxivity (*R*_2_) in units of mM^−1^ s^−1^. (**b**) Signal intensity from MION phantoms over a range of 0–7 mg/ml (based on iron content) demonstrating CT contrast with gold (top). CT contrast, measured in Hounsfield units (HU), was calculated for each sample and were plotted versus iron concentration (bottom).

Next, we look at the impact of the silica and gold coating on the functionality of the nanoparticles, beginning with imaging (both MRI and x-ray CT contrast). Using *T*_2_-weighted magnetic resonance sequences, MR contrast capabilities of the MION series (**1**, **2** and **3)** were compared (Fig. 4a). As expected, *T*_2_ relaxivity increased linearly with iron concentration, which is consistent with reported observations of JHU MIONs^49^. This was true for all three constructs, and again supports the magnetometry results which indicate minimal change to the magnetic properties. Furthermore, of the MION and coated MION samples prepared and tested, only AuSi-MIONs demonstrated significant concentration-dependent signal intensity with CT at concentrations relevant for therapy. At a solution concentration of ~7 mg Fe/mL, the signal intensity of the AuSi-MIONs was ~360 Hounsfield units (HU) (Fig. 4b), supporting our hypothesis that a gold coating provides complementary CT contrast to MRI. For reference, water is assigned a value of 0 HU, and clinical contrast agents typically measure >100 HUs. The JHU MIONs and Si-MIONs were typically <100 HU.

Adding x-ray opacity to magnetic iron oxide nanoparticles has significant benefit for imaging-guided therapy (hyperthermia) applications because tissue concentrations required, >1 mg Fe/g tissue, often produce artifacts with magnetic resonance which is more sensitive to the magnetic moments of magnetic iron oxide nanoparticles making it difficult to reliably image tissue concentrations >0.1 mg Fe/g tissue. A magnetic iron oxide construct having both x-ray opacity and significant responsiveness to an alternating magnetic field provides significant benefit for imaging-guided magnetic hyperthermia.

**Figure 5:**
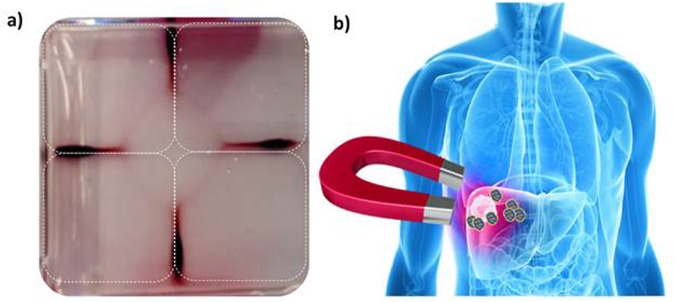
Schematic of magnetic vectorization, and nanoparticle accumulation in response to static field. (**a**) Photograph showing AuSi-MIONs drawn by four permanent magnets (dotted outlines) demonstrating potential for magnetic localization. (**b**) Illustration of potential for magnetic localization. Images of liver cancer and big red magnet are used with permission from Dreamstime.com LLC.

The responsiveness of MIONs to magnetic fields contributes to their therapeutic potential as well as imaging. This property provides several routes to enhance therapy by enabling remote localization (conceptually illustrated in Fig. 5b) of nanoparticles *in vivo* through placement with external static (time-invariant) gradient magnetic fields, or by generating heat when a region containing MIONs is exposed to alternating magnetic fields (AMFs). AuSi-MIONs (purple) show potential for use in magnetic location control because they migrate when exposed to a permanent (static) magnet (Fig. 5a).

**Figure 6:**
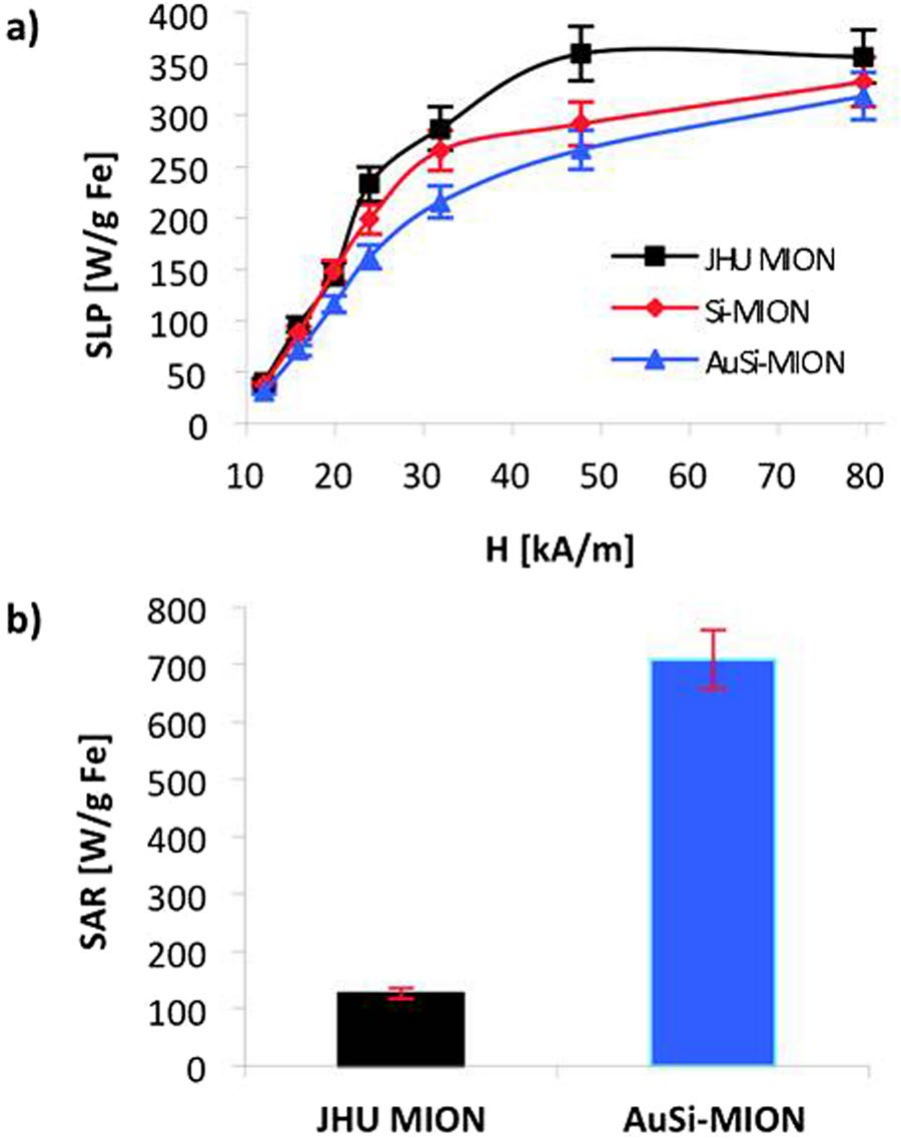
SLP in AC magnetic field and SAR in laser. (**a**) Specific loss power (SLP), a measure of heating efficiency in an alternating magnetic field, was measured for JHU MIONs (square), Si-MIONs (diamond) and AuSi-MIONs (triangle) at a frequency of 150 kHz ± 5 kHz over a range of amplitudes from 10 to 80 kA/m. (**b**) Comparison of laser-induced heating, reported as specific absorption rates (SARs, normalized by iron content) between JHU MIONs and AuSi-MIONs. A 5.5 W laparoscopic laser was centered on each solution for 15 seconds. The change in temperature was monitored and SARs were calculated for each sample.

Magnetic nanoparticle-mediated hyperthermia can be used to treat deep-tissue tumors by remote activation of MIONs with AMFs, which are not attenuated by tissue^8^. On the other hand, AMF-induced power deposition in tissue can occur, generating off-target heating if large volumes are exposed to AMFs having high frequency and amplitude^8,54^. JHU MIONs demonstrated suitable heating at low-amplitude (<10 kA/m peak) AMFs (Fig. 6a), measured using methods previously reported^48,49,55^. Coating with gold had only a modest effect on the measured heating efficiency (Fig. 6a).

Gold-shell nanoparticles have demonstrated non-radiative heating potential via surface plasmon resonance when excited by a laser^35,56,57^. This mechanism of heating differs from magnetic nanoparticle-mediated heating, offering alternate therapeutic options with gold-coated MIONs. Laser heating of JHU MIONs and AuSi-MIONs was compared using a 5.5 W laparoscopic laser (780 nm) by illuminating MION-containing solutions and measuring temperature with optical fiber temperature probes (Fig. 6b). The specific absorption rates (SAR) of AuSi-MIONs (709 Wg^−1^ Fe) and JHU MIONs (127 Wg^−1^ Fe) were estimated after normalizing by iron content^55^. Comparison of these results confirms that the laser-induced temperature increase is significantly enhanced with gold coating, although the iron oxide core can generate modest heat when exposed to laser energy. This provides additional evidence of the continuity of the gold coating.

**Figure 7:**
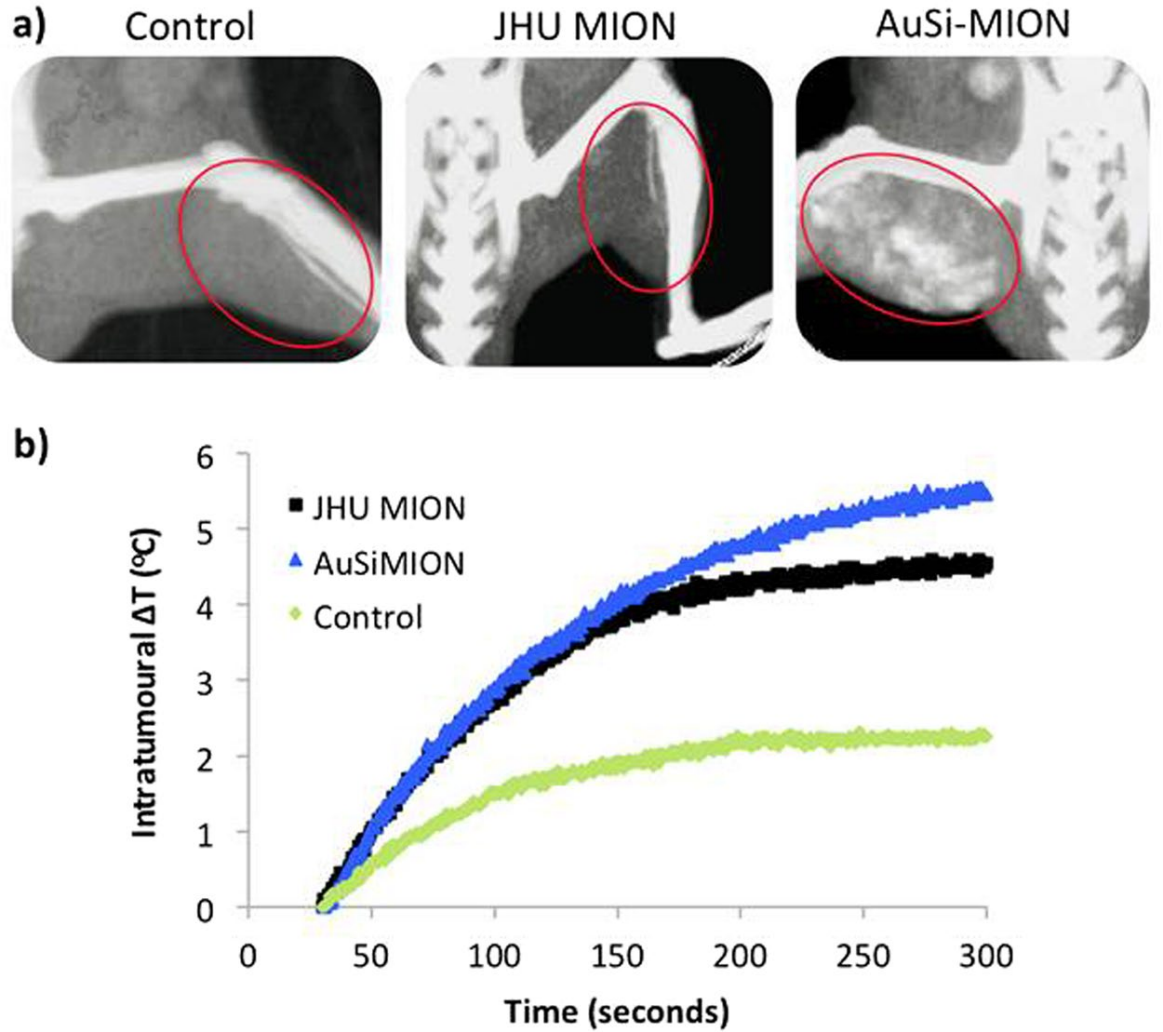
*In vivo* imaging and heating. (**a**) *In vivo* CT imaging in nude male mice bearing human prostate (LAPC-4) cancer xenograft tumors. Control: saline only injection (left) - the red oval denotes the location of the tumor, which is invisible without added contrast. JHU MIONs (middle): injection concentration of 5.5 mg Fe/cm^3^ tumor. Iron oxide demonstrates insufficient x-ray contrast with CT rendering the tumor invisible. AuSi-MIONs (right): injection concentration of 5.5 mg Fe/cm^3^ tumor. The AuSi-MIONs are visible in the tumor indicated by increased signal. (**b**) Following CT imaging, mice were placed in an AMF device (150 kHz, 40 kA/m) and a ~6°C rise of tumor temperature was measured with RF-resistant optical fiber temperature probe inserted into tumors loaded with either JHU MIONs or AuSi-MIONs.

**Figure 8:**
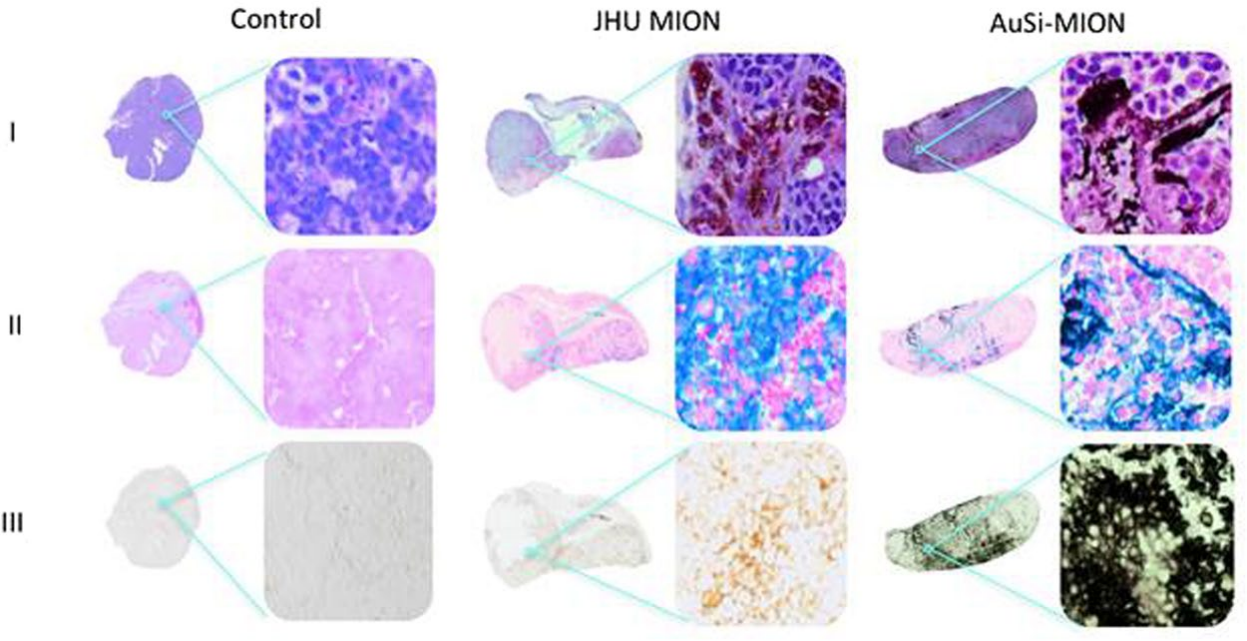
Histology of prostate tumor xenografts. (**a**) Mice were euthanized and tumor tissues were collected for staining 72 h post AMF exposure (Row I: H&E, row II: Prussian blue and row III: silver enhancement stain). The control shows no iron oxide or gold present. Tissues from the mouse injected with JHU MIONs show iron oxide particles in the H&E stain, iron staining (blue) with Prussian blue and no response to the silver enhancement stain. Tissues from the mouse injected with AuSi-MIONs show a dark purple color from the gold nanoparticles in the H&E stain and iron staining (blue) with Prussian blue. Dark black staining of the AuSi-MIONs by the silver enhancement stain, which only stains metallic gold or silver, can be seen in row III. Whole tumor images are composites created from separate 4x images; magnified images were obtained at 20x.

To complement the *in silico* measurements, we conducted pilot *in vivo* tests. CT imaging and magnetic heating were assessed in mice bearing subcutaneous LAPC-4 human prostate tumor xenografts on their thigh (Fig. 7). Mice received intra-tumor injections of either MION or saline (negative control) solutions. CT contrast of tumors injected with AuSi-MIONs was enhanced and consistent with results obtained from gel phantoms, whereas neither the saline nor JHU MIONs provided measurable contrast (Fig. 7a). AuSi-MIONs remained in the vicinity of the tumor for up to 13 days, as determined in a fourth mouse (Fig. S2). Following imaging with CT, mice were exposed to an AMF using methods described previously^48,58^. Tumors injected with either nanoparticle construct heated by 4°C within 2–3 min (Fig. 7b) of exposure to AMF, while only minor heating due to eddy currents, occurred in the tumor injected with saline control. Following AMF therapy, mice were sacrificed and tumors were excised and processed for histology. Histological examination of tumors stained with hematoxylin and eosin (H&E) (Fig. 8) revealed that the JHU MIONs (brown) and the AuSi-MIONs (purple) could be visualized (row I), although confirmation from more specific stains was needed. Stains for iron oxide and gold deposits in tissue, Perls’ (Prussian blue) reagent (row II) and silver enhancement (row III), respectively, confirmed the presence of those species in tumor tissue samples. We note that the AuSi-MION tumor sample also stained blue with Perls’ reagent, perhaps because sample preparation degraded the gold coating, exposing iron oxide. On the other hand, only the sample obtained from tumor injected with AuSi-MION stained positive (black color) with silver enhancement stain (row III), demonstrating the presence of gold^59^. Further detailed characterization is needed with additional numbers of animals to confirm therapeutic potential of heating and correlate quantitative analysis with imaging and histology.

In summary, we report the synthesis and physical characterization of a magnetic iron oxide nanoparticle construct in which magnetic properties of iron oxide cores are not unduly impacted, and new functionality is added by silica then gold coating to achieve multi-modal imaging and heating capability using a single nanoparticle system. Comprehensive physico-chemical characterization with multiple techniques, including SANS, DLS, TEM, and magnetometry, confirmed a continuous gold layer and revealed unexpected silica intercalation with the dense polycrystalline MION cores. This silica intercalation promoted formation of a continuous gold coating while preserving the magnetic behavior. The sensitivity of magnetic resonance can be used to detect low concentrations of the Au-Si-MIONs in tissue, while the gold shell enables x-ray visualization of higher nanoparticle concentrations needed for therapeutic applications. Magnetic properties sufficient for remote localization with gradient magnetic fields and heating with alternating magnetic fields were demonstrated, as well as additional heating capability with laser activation. This work demonstrates for the first time a continuous gold-silica coating of magnetic iron oxide nanoparticles in which the magnetic properties are preserved sufficient to retain significant hysteresis heating capability. We also note the critical need for accurate and correct physical and magnetic characterization of nanostructured materials, free of assumptions typically encountered when coating. The identification of the key structural characteristics responsible for the robust performance of this MION formulation can be exploited for future applications and development.

## Experimental Methods

We identify certain commercial equipment, instruments, or materials in this article to specify adequately the experimental procedure. In no case does such identification imply recommendation or endorsement by the National Institute of Standards and Technology, nor does it imply that the materials or equipment identified are necessarily the best available for the purpose.

### Materials for syntheses

Tetraethylorthosilicate (TEOS), tetrakis (hydroxymethyl) phosphonium chloride (THPC), 3-aminopropyltrimethoxysilane (APTMS), sodium hydroxide, hydroxylamine (50% in H_2_O), potassium carbonate and chloroauric acid tetrahydrate (HAuCl_4_^.^4H_2_O) were obtained from Sigma-Aldrich. Ammonium hydroxide solution (30%) was purchased from Merck Company. All the reagents were analytical grade and used as received.

### Synthesis of gold colloid suspension

Aqueous sodium hydroxide (1 M, 600 mL) and aqueous THPC (1.2 mM, 2 mL) were added to 90 mL of deionized water and stirred rapidly for ten minutes. Chloroauric acid (1 wt%, 3.4 mL) was quickly added and the solution immediately turned dark brown. The solution was stored at 4 °C.

### Synthesis of AuSI-MIONs

JHU MIONs were coated with silica using a modified Stöber method^52^. JHU MIONs and 30% ammonium hydroxide were added consecutively to a solution of ethanol and water. The nanoparticle mixture was sonicated for 15 minutes followed by addition of TEOS and the flask was placed on a mechanical rocker overnight. The silica-coated particles were washed three times with ethanol by centrifugation to remove excess TEOS. The particles were redispersed in water, APTMS was added, and the solution was mixed overnight on a rocker. Amino-terminated nanoparticles were washed three times in ethanol by centrifugation. The silica surface was seeded with the gold THPC colloid suspension (See Supporting Information). The THPC precursor solution was diluted with aqueous K_2_CO_3_ and sonicated for two minutes. Aqueous sodium chloride, 1 M (molL^−1^) and amino-terminated nanoparticles were added to the solution and sonication continued for two minutes. The solution was stored overnight at 4 °C. Gold seeded nanoparticles were washed once by centrifugation with aqueous K_2_CO_3_ and three times using a permanent magnet. The particles were redistributed in aqueous K_2_CO_3_, followed by addition of 1% HAuCl_4_ solution. The solution was mixed by vortexing for 30 min and hydroxylamine (50% in H_2_O) was added. The mixture immediately turned dark purple. The nanoparticles were washed three times with aqueous K_2_CO_3_ using a permanent magnet, resuspended in aqueous K_2_CO_3_ and stored at 4 °C.

### Nanoparticle characterization

A summary of samples prepared and characterization performed is provided in Table 1.

#### Dynamic light scattering (DLS)

The hydrodynamic diameter of the citrate-stabilized (uncoated) Fe_3_O_4_/γ-Fe_2_O_3_ and coated (Si or Au-Si) nanoparticles was measured on a Zetasizer Nano (Malvern Instruments, Worcestershire, UK) in 1.8 mM K_2_CO_3_^58^ with 0.01 wt % nanoparticles. A refractive index of 1.33 (Fe_3_O_4_) and 2.42 for DI water were used, and instrument setting was volume mode.

#### Transmission electron microscope (TEM)

A Philips EM 420 transmission electron microscope was used to acquire images^58^. Samples were prepared using 10 μL of nanoparticle suspension in 100 μL of water placed on a carbon coated copper grid (Ted Pella, Inc., Redding, CA)^58^, and dried for 24 hrs at room temperature. Note that images shown were taken of individual nanoparticles well separated from any large clusters.

#### Magnetometry

Measurements were performed at 300 K from ± 5,570 kA/m (±70,000 Oe) and at 5 K from ± 5,570 kA/m (±70,000 Oe) using a superconducting quantum interference device with vibrating sample magnetometer (SQUID VSM) (Quantum Design, Inc). The 5 K data were measured after cooling either in zero applied magnetic field or in the presence of an applied magnetic field of 5.6 MA/m (70,000 Oe). Samples were loaded into Kel-F liquid capsules (LakeShore Cryogenics), and sealed with epoxy to preserve water during measurement under vacuum. Data are normalized to either total iron content, determined by ferene-s method previously described^60^, or by total solid content.

#### Small angle neutron scattering (SANS)

Unpolarized SANS data were acquired on the CHRNS 30 m SANS (NG7) instrument at the National Institute of Standards and Technology Center for Neutron Research (NCNR) in Gaithersburg, Maryland. Neutron wavelength was 0.84 nm in transmission. Instrument configurations enabled measurements having scattering vectors (*Q*) from 3 × 10^−5^ to 5 × 10^−1^ Å^−1^ using three detector settings (15 m, 4 m and 1.33 m). Samples were measured in water (H_2_O) at room temperature.

The raw 2D data obtained from the SANS experiments were reduced via the software program Igor Pro^53^ in order to provide the corrected 1D data. The raw 2D data were corrected for empty cell, solvent, and background scattering (blocked beam) as well as detector non-uniformity. The data were plotted as *I* vs. *Q* with circular averaging of data at each value of scattering vector, *Q*. The 1D *I*(*q*) data obtained from each instrument configuration were combined into a single file spanning the three *q* ranges. In order to combine the data from all three *q*-ranges, the data sets were normalized by absolute intensity and combined using established procedures^51,53^. The result was a complete representation of the scattering from each sample over the range from *Q* = 3 × 10^−5^ to 5 × 10^−1^ Å^−1^. A variety of (geometrical) model functions were used to fit to the entire scattering curve obtained from each sample with SasView (NIST, Gaithersburg, MD)^53,61^. It was immediately evident that no single geometrical model was adequate to analyze the full SANS data obtained from each sample (Fig. S1a); the complex structure of the nanoparticles required a linear combination of multiple model functions, which was accomplished using SasView (Supplemental Information). Model fitting was constrained using data from other measurements, e.g. DLS and by combining known material properties, e.g. scattering length density. Furthermore, the models were correlated between samples by using the parameters determined in the previous model (e.g., using the JHU-MIONs crystallite sizes in the Si-MIONs fits), so that subsequent models were constrained by the earlier results.

#### T2-weighted magnetic resonance imaging

*T*_2_-weighted images of gel phantoms containing MIONs ranging from 0 to 80 μg Fe/ml were obtained using a Bruker 9.4 T horizontal bore spectrometer using spin-echo sequence parameters: repetition time (TR) = 4000 ms, echo time (TE) = 4, 8, 12, 16, 20, 24, 28 and 32 ms, slice thickness = 40 mm, resolution = 128 × 128 pixels^58^. Images were reconstructed and analyzed with ImageJ (NIH, Bethesda, MD) software. To assess the MR contrast capabilities of MIONs **1**, **2** and **3**, phantoms ranging in iron concentration from 0–80 μg/ml (0–1.4 mM) were imaged. The graph inset shows iron concentration (mM) plotted versus the inverse of *T*_2_. The trendline slopes for each nanoparticle give *R*_2_, the transverse relaxivity coefficient, which is a measure of nanoparticle contrast efficiency. The *R*_2_ values were 155, 99 and 68 mM^−1^ s^−1^ for MIONs **1**, **2** and **3**, respectively.

#### Histology and confocal microscopy

Following CT imaging and/or AMF hyperthermia therapy, mice were sacrificed and tumors were excised. Tumors were fixed for at least 48 hours in 10% formalin solution before being embedded in paraffin. The paraffin blocks were sectioned and stained with hematoxylin and eosin (H&E), Prussian blue, or silver enhancer. H&E and Prussian blue staining were performed by the Molecular & Comparative Pathobiology Histology Core at Johns Hopkins Medical Institute. The silver enhancement kit was used according to the kit instructions (BBI Solutions, Cardiff, UK). The histological sections were examined under an Eclipse 80i microscope (Nikon Instruments, Inc., Melville, NY). Whole-slice images were assembled from multiple images obtained at 4x magnification. Magnified images were obtained with a 20X objective.

#### Laser heating

Heating rates of JHU MIONs and AuSi-MIONs via laser excitation were compared in solution using a 5.5 W (780 nm) laparoscopic laser directed at the nanoparticle solutions. The increases in temperature were monitored using a FLIR thermal imaging camera and SARs were normalized based on iron content.

#### X-ray computed tomography imaging

X-ray computed tomography (CT) imaging was performed on gel samples loaded with nanoparticle concentrations ranging 0–7 mg Fe/ml. CT imaging was performed at 65 kV and 0.7 mA with a SARRP (xStrahl Ltd., Surrey, UK) system. Images were reconstructed using 1800 projections and Hounsfield units were calculated for each nanoparticle concentration with ImageJ software.

#### Alternating magnetic field (AMF) equipment and specific loss power (SLP) measurements

The AMF system and SLP measurements have been previously described in detail^55,62–64^. SLP values were estimated from heating data using the relationship SLP = ${C/{m}_{Fe}(dT/dt)|}_{t\to 0}$, where *m*_*Fe*_ is the mass of iron in the sample for iron oxide-based nanoparticles; *C* is the heat capacity of the sample (assumed to be that of water or 4.18 J/g °C); and, ${\rm{\Delta }}T/{\rm{\Delta }}t$ is the measured rate of temperature rise (Δ*T*) during the heating interval (Δ*t*) or T_n_ − T_n−1_ vs. Δt^55,64^.

#### Pilot in vivo studies

Four male nude mice (Hsd: Athymic Nude-Foxn1^nu^, Harlan Labs, Indianapolis, IN) were used in this study. All were 5 to 7 weeks old and weighed about 20 grams prior to treatment with AMF and nanoparticles. Mice were housed in an Association for Assessment and Accreditation of Laboratory Animal Care (AAALAC)-accredited facility in compliance with the Guide for the Care and Use of Laboratory Animals^65^, and procedures were approved by the Johns Hopkins Institutional Animal Care and Use Committee (IACUC). Male nude mice were selected for their relevance to our ongoing studies on prostate cancer therapy. Mice bearing human prostate tumor xenografts were anesthetized using an isofluorane chamber and maintained under anesthesia using a nose cone. Following a pre-injection CT image obtained at 65 kV and 0.7 mA using the Small Animal Radiation Research Platform (SARRP)^66^, study mice were injected with either JHU MIONs or AuSi-MIONs to a target iron concentration of ~5.5 mg Fe/cm^3^ tumor^48,67^. Saline control intratumor injection was a comparable volume ~20 μL as used for MION injections. A second CT image was immediately acquired post-injection of the nanoparticles or saline, and the image was reconstructed using ImageJ software. For hyperthermia, equipment and methods were used as described previously^48,58,67^. The AMF system was adjusted for stable oscillation at 150 ± 5 kHz and 40 kAm^−1^ peak amplitude. Intratumor, rectal, and contralateral skin temperatures were monitored with fiber optic temperature probes (FISO, Inc., Quebec, Canada)^60^. Heating was conducted for 20 minutes.

## Electronic supplementary material

Supporting Information
