# Supplementary material for: Nanoparticle architecture preserves magnetic properties during coating to enable robust multi-modal functionality
Source: Sci Rep. 2018 Aug 23;8:12706. doi: 10.1038/s41598-018-29711-0 (PMC6107675; doi:10.1038/s41598-018-29711-0)
Supplement: Supplementary file 1 — Supporting Information [file 41598_2018_29711_MOESM1_ESM.docx]

Supporting Information

**Nanoparticle architecture preserves magnetic properties during coating to enable robust multi-modal functionality**

Lauren E. Woodard, Cindi L. Dennis, Julie A. Borchers, Anilchandra Attaluri, Esteban Velarde, Charlene Dawidczyk, Peter C. Searson, Martin G. Pomper and Robert Ivkov*

**Small Angle Neutron Scattering (SANS) Analysis**

The scattering geometry for all our SANS measurements is shown in Fig. S1A. The 2D scattering for each sample (e.g., Figure S1B) was reduced to 1D by performing a circular average, as described in the Methods Section. Fitting was guided by nanoparticle dimensions obtained from complementary measurements (DLS and TEM). DLS is a useful technique for sizing nanoparticles in suspension; however, it provides only a hydrodynamic diameter (which can differ significantly from the inorganic physical dimensions if there is a polymer coating). DLS also assumes that particles are spherical, and larger particles dominate DLS because smaller particles scatter less light. TEM reveals that the complex nanoparticles have distinct features [e.g., core and outer shell(s)] of differing size that are expected to produce scattering in different Q ranges. Best fits to the SANS data for each sample (Fig. S2) thus included a summation of two standard SANS models (Figure S1C, S1D, and S1E) that were empirically determined to best capture the dominant features at high and low *Q*. Structural features common to both models (such as material scattering length densities) were constrained for consistency. Furthermore, known parameters were constrained (such as the crystallite size) when adding a new layer. Note attempts to fit these data with a single SANS model (Fig. S3) were unsuccessful.

Considering first the JHU MIONS, TEM images [Figure 2c (1)] showed size (and some shape) dispersity. We therefore hypothesized that a triaxialellipsoid model (Fig. S1D) was appropriate for fitting to the SANS data. It was also determined that some “flat” JHU MIONs tend to stack in solution, giving rise to a sharply increasing tail in the low-*Q* region. JHU MION scattering data were thus modelled (Figures 3a and S2A) by summing triaxialellipsoid and stacked discs models (Figures S1C and S1D, respectively). It is to be noted that neither the triaxialellipsoid model nor the stacked discs model are able to fit the data on their own (Figure S3A). Dimensions and 3D-representations of the summed fitting analysis can be seen in Figure 3b, and the lengths of the three elliptical axes of the JHU MION were found to be 11, 39 and 78 nm. Note that the smallest dimension is consistent with the approximate diameter of one or two of the iron oxide crystallites within the core. We can thus account for the discrepancy between DLS and SANS size measurements because weighted DLS measurements (55 nm) favor the average of the triaxialellipsoid dimensions (43 nm). The fitted SANS value for the core MION scattering length density (SLD) is 8.2 x 10^-6^ Å^-2^, which is consistent with the theoretical SLD of magnetite 6.9 x 10^-6^ Å^-2^ and the theoretical magnetic SLD of magnetite 1.4 x 10^-6^ Å^-2^, the sum of which equals 8.3 x 10^-6^Å^-2^.

Guided again by TEM measurements [Fig. 2c(2)], SANS data obtained from the Si-MION sample were fitted (Figures 3a and S2B) by summing a triaxialellipsoid model (Fig. S1D) with a core-shell sphere model (Fig. S1E). It is worth noting again that neither triaxialellipsoid nor the core-shell models could singularly describe the data (Figure S3B). The overall dimensions of the particles with the outer silica shell were determined by the triaxialellipsoid model and were found to be 12, 53 and 137 nm with an SLD of 4.3 x 10^-6^ Å^-2^. DLS measurements (81 nm) which assume a spherical particle shape are again in reasonable agreement with the average of the triaxialellipsoid dimensions (67 nm). The additional deviation is likely due to the change in refractive index of the MION close to the surface impacting the model assumptions. The core-shell sphere model was consistent with TEM images of Si-MIONs, which show that each iron oxide crystal making up the core appears to be coated with a thin layer of silica, which then provides greater contrast for SANS measurements. Analysis of the core-shell yielded dimensions of 6 nm for the diameter of each magnetite crystallite (SLD = 7.4 x 10^-6^ Å^-2^) and an average of 2 nm of silica (SLD = 4.3 x 10^-6^ Å^-2^) surrounding each crystal. The core SLD decreased slightly compared to that of the bare JHU MION cores. The decrease can be explained by the presence of a mixed iron oxide/silica layer between the iron oxide core and silica shell. Such intermixing has potential to produce an iron silicate which may possess an altered micromagnetic structure, depending on the surface chemistry of the iron oxide at the time of coating.

Consistent with the TEM analysis [Fig. 2c(3)], AuSi-MION SANS data (Figures 3a and S2C) were also fitted with a summation of the triaxialellipsoid (Fig. S1D) and core-shell sphere (Fig. S1E). The dimensions of the triaxialellipsoid increased to 21, 117 and 299 nm with the average SLD of the ellipsoid decreasing slightly to 4.1 x 10^-6^ Å^-2^. This decrease in SLD with the addition of the gold is expected given the theoretical SLD of gold is slightly lower than that of silica (4.2 x 10^-6^ Å^-2^ and 4.5 x 10^-6^ Å^-2^, respectively). Again, DLS measurements (145 nm) agreed with the average of the triaxial ellipsoid dimensions (146 nm). The diameter of the core-shell sphere remained the same as in the Si-MION sample (6 nm), indicating that the internal iron oxide crystallites were unaffected by the gold-plating process. The thickness of the shell, which now includes both silica and gold, increased the total core-shell sphere diameter to ~13 nm.


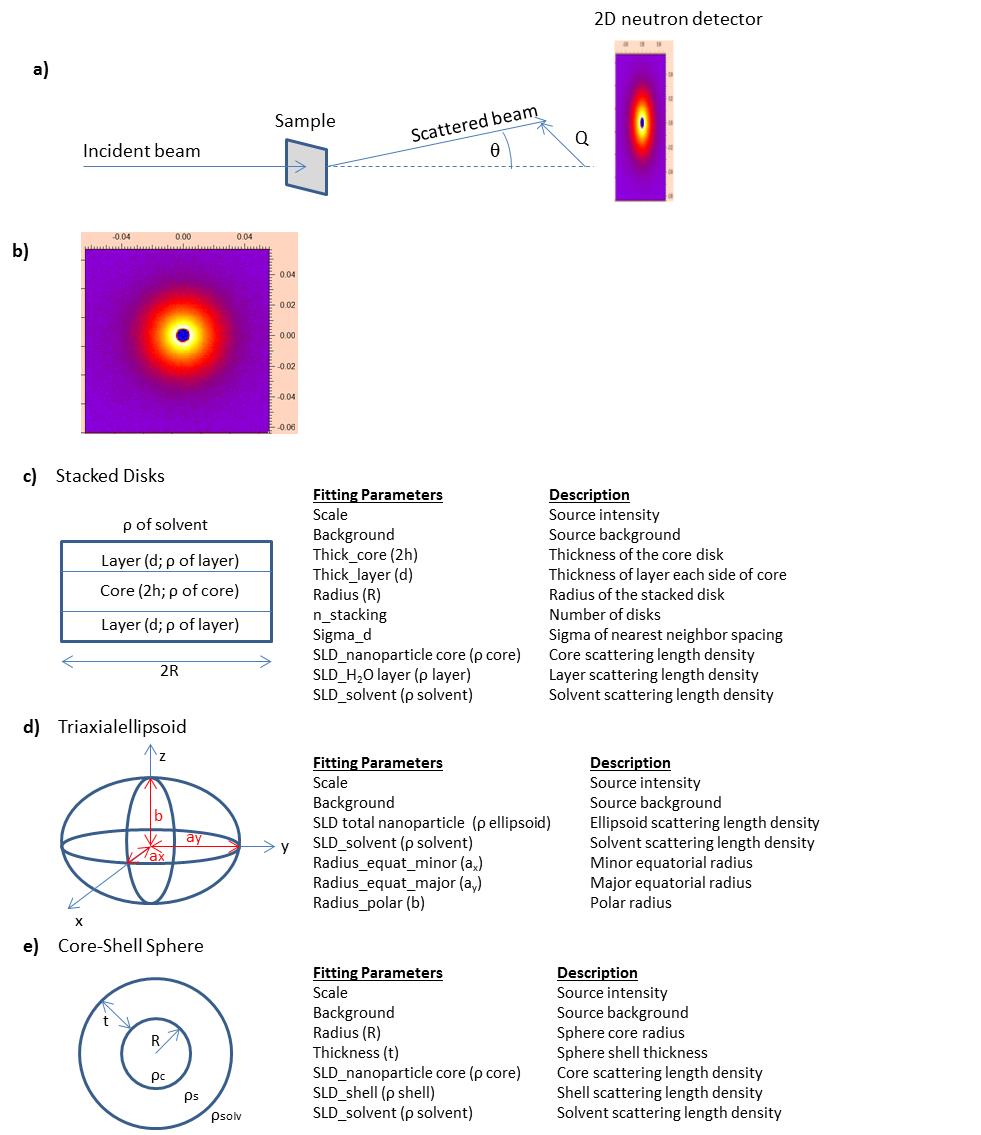


**Figure S1:** Schematics of a) small angle neutron scattering (SANS) experimental configuration showing a representative 2-D scattered neutron intensity as captured by the 2-D detector; b) representative image of scattered intensity; c) stacked discs model with list of fitting parameters; d) triaxialellipsoid model with fitting parameters; and, e) core-shell sphere model with fitting parameters that were used to fit the SANS data. Fitting parameters were either fixed or constrained within limits when measured independently by complementary techniques. Additional information about the models is provided by the NIST Center for Neutron Research (NCNR) at:

<http://www.sasview.org/docs/user/index.html>

<https://www.ncnr.nist.gov/programs/sans/data/Download/SANS_Model_Docs_v4.00.pdf>

**C**

**B**

**A**

**Figure S2.** Results obtained from fitting summed geometrical models to SANS data. Models included ‘stacked discs’ + ‘triaxialellipsoid’ for JHU MIONs; and, ‘core-shell’ + ‘triaxialellipsoid’ for Si-MIONs and AuSi MIONs. Note that individual geometrical models failed to adequately fit the entire data range. When combined, however summed models provided adequate fits to the data over the entire range (see manuscript text).


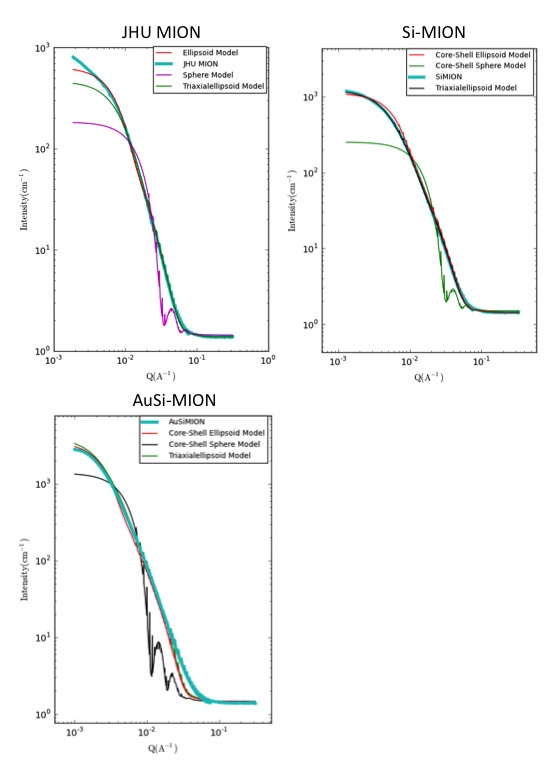


**C**

**B**

**A**

**Figure S3.** Results obtained from fitting only individual geometrical models to SANS data.

**C**

**Magnetometry Analysis**

Further evidence of the silica intercalation around the iron oxide crystallites that form the dense polycrystalline core was found in earlier samples (using the sample JHU-MIONs as starting material), which were used to develop the silica coating. Specifically, this development sample series examined the impact of growing the silica shell, from a “partial” coating (with no measurable change in the DLS hydrodynamic diameter), to a 178 nm thick coating (as determined by DLS). Only the first two “complete” coatings (67 nm and 78 nm) displayed a zero magnetic field loop shift (Fig. S7) . This was absent in the uncoated, precursor JHU-MIONs, thus we conclude it was a direct result of the silica coating. Fe and Si are known to react to form various types of silicides, and the presence of oxygen only complicates the temporary structures that can form. Thicker layers are likely to complete the oxidation of the silica, preventing the formation of these complex silicides with thicker coatings. However, an outer core surface coating (or partial coating) is unlikely to produce enough pinned/uncompensated spins at the surface or exchange bias to generate a measurable effect, given the total volume of the core. Conversely, if the silica is intercalating around the iron oxide crystallites (creating uncompensated/pinned spins at the surface of the crystallites) then the volume affected would be substantially greater (compared to the total volume and in absolute terms) and likely measurable.

**

**

**Figure S5**. Magnetic characterization of JHU-MIONs as a function of coating type when normalized to total iron content (as determined by ferrozine assay). Measurements were performed at 300 K. There is a measurable decrease in the saturation magnetization with silica and gold coating due to diamagnetic shielding.





**Figure S6**. Magnetic characterization of JHU-MIONs as a function of coating type when normalized to total solid content. Measurements were performed at 5 K after zero field cooling. There is a measurable increase in the coercivity with silica coating due to immobilization of the cores that disappears with the additional gold coating.


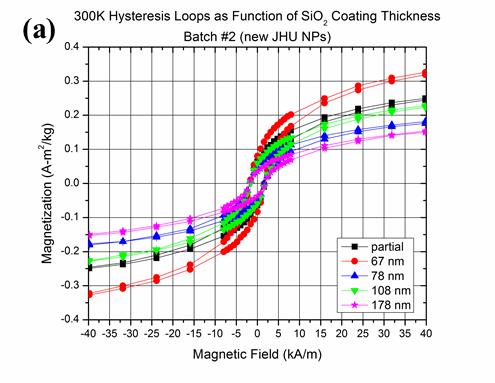


**
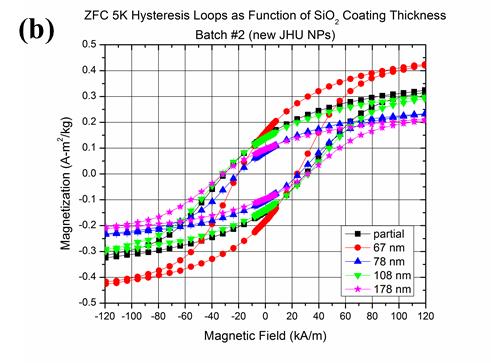
**

**
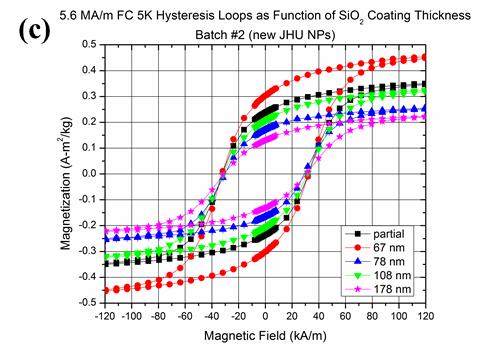
**

**Figure S7**. Magnetic characterization of JHU-MIONs in silica coating development series both as a function of coating thickness (determined by difference between hydrodynamic radius from DLS before coating and after coating). Measurements were performed at (a) 300 K and (b) 5 K after zero field cooled and (c) 5 K after field cooling. Only the first two “complete” silica coatings (Si-MIONs with average diameters of 67 nm and 78 nm) displayed zero field magnetic loop shifts due to uncompensated/pinned spins at the surface of the crystallites due to silica intercalation.

**X-ray imaging**


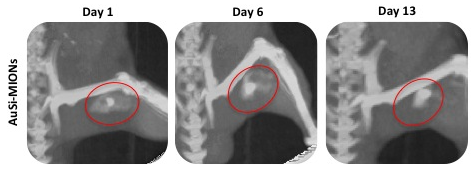


**Figure S8.** CT monitoring of AuSi-MION location in LAPC-4 model (red oval denotes tumor location). Following intratumoral injection of AuSi-MIONs (5.5 mg iron oxide per cm^3^ of tumor) into the hind leg of a nude mouse, signal intensity was monitored over 13 days. CT scans were performed immediately following the injection of particles, on day 6 and on day 13. Particles were still clearly visible on day 13 with no decrease in signal intensity.
